# Emerging roles of FOXK2 in cancers and metabolic disorders

**DOI:** 10.3389/fonc.2024.1376496

**Published:** 2024-04-29

**Authors:** Xiaoke Xing, Xiangyong Que, Sihao Zheng, Shuxian Wang, Qibin Song, Yi Yao, Pingfeng Zhang

**Affiliations:** Cancer Center, Renmin Hospital of Wuhan University, Wuhan, China

**Keywords:** FOXK2, cancer, chemotherapy resistance, post-translational modifications, *de novo* nucleotide synthesis, metabolic disorders

## Abstract

FOXK2, a member of the Forkhead box K (FOXK) transcription factor family, is widely expressed in various tissues and organs throughout the body. FOXK2 plays crucial roles in cell proliferation, differentiation, autophagy, *de novo* nucleotide biosynthesis, DNA damage response, and aerobic glycolysis. Although FOXK2 is recognized as an oncogene in colorectal cancer and hepatocellular carcinoma, it acts as a tumor suppressor in breast cancer, cervical cancer, and non-small cell lung cancer (NSCLC). This review provides an overview of the recent progress in understanding the regulatory mechanisms of FOXK2 and its downstream targets, highlights the significant impact of FOXK2 dysregulation on cancer etiology, and discusses the potential of targeting FOXK2 for cancer treatment.

## 1 Introduction

**Figure 1:**
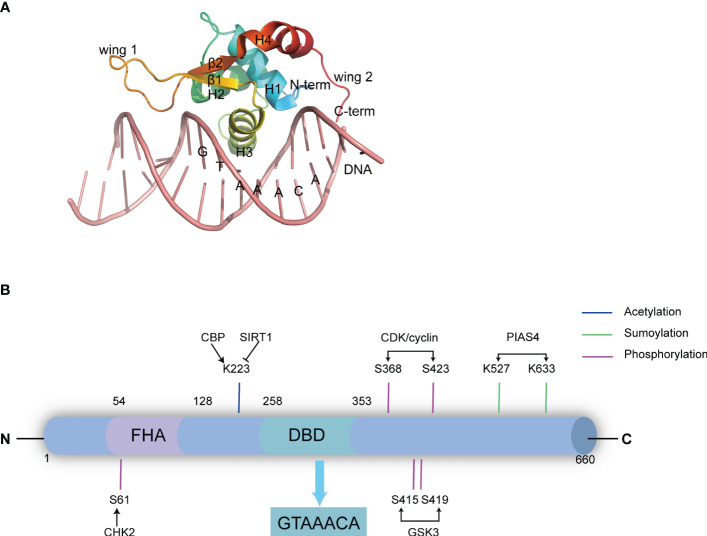
The domain structure and post-translational modification sites of FOXK2. **(A)** The DBD domain of FOXK2 and its interaction with DNA (derived from PDB code: 2C6Y). The secondary structural elements in DBD (β1-β2, H1-H4), the N-terminal (N-term) and the C-terminal (C-Term), and the wing1 and wing2 regions are labelled. Additionally, the binding DNA with the consensus sequence is shown. **(B)** FOXK2 consists of 660 amino acids, including a characteristic FHA domain and DBD domain. The DBD domain binds the GTAAACA sequence in DNA promoter regions of its target genes. Notably, FOXK2 undergoes various post-translational modifications. CHK2 catalyzes phosphorylation at S61, GSK3 catalyzes phosphorylation at S415 and S419, CDK/cyclin phosphorylates S368 and S423, PIAS4 catalyzes SUMOylation at K527 and K633, while CBP acetylates K223. Additionally, SIRT1 catalyzes deacetylation of FOXK2.

The Forkhead box (FOX) family comprises 19 subfamilies (from FOXA to FOXS) of highly conserved transcription factors, characterized by a highly conserved winged-helix (also called forkhead motif) DNA binding domain (DBD) containing four α-helices and two β-strands flanked by two wings (**Figure 1A**). The DBD domain interacts with DNA through the binding of the third α-helix into the DNA major groove, while the two flanked wings bind to the DNA minor groove (**Figure 1A**) (1–3). Each FOX protein, while sharing the common DBD domain, possesses distinct additional domains, determining their specific functions. For instance, FOXA possesses a transactivation domain (TAD) in addition to the common DBD domain. This feature makes FOXA a pioneer transcription factor, facilitating the recruitment of other transcription factors to their target genes, particularly nuclear receptors such as androgen receptor (AR) (4), glucocorticoid receptor (GR) (5) and estrogen receptor (ER) (6, 7). Subsequently, this establishes FOXA a close association with tumorigenesis. FOXM has a N-terminal repressor domain (NRD) that directly interacts with its C-terminal TAD, thereby weakening its transcriptional activity; which enables it to play crucial roles in controlling the cell cycle (8). FOXO possesses a nuclear export signal (NES) and a nuclear localization signal (NLS), which are closely related to the regulation of FOXO nuclear localization and export by AKT and 14-3-3 proteins, thereby affecting the transcriptional regulation of FOXO on cell cycle, apoptosis and cell metabolism (9). FOXP is very different from other FOX members, it contains a proline-rich (Pro-R) domain at the N-terminal, which recruits class I HDACs thus functions as a transcriptional repressor (10).

**Figure 2:**
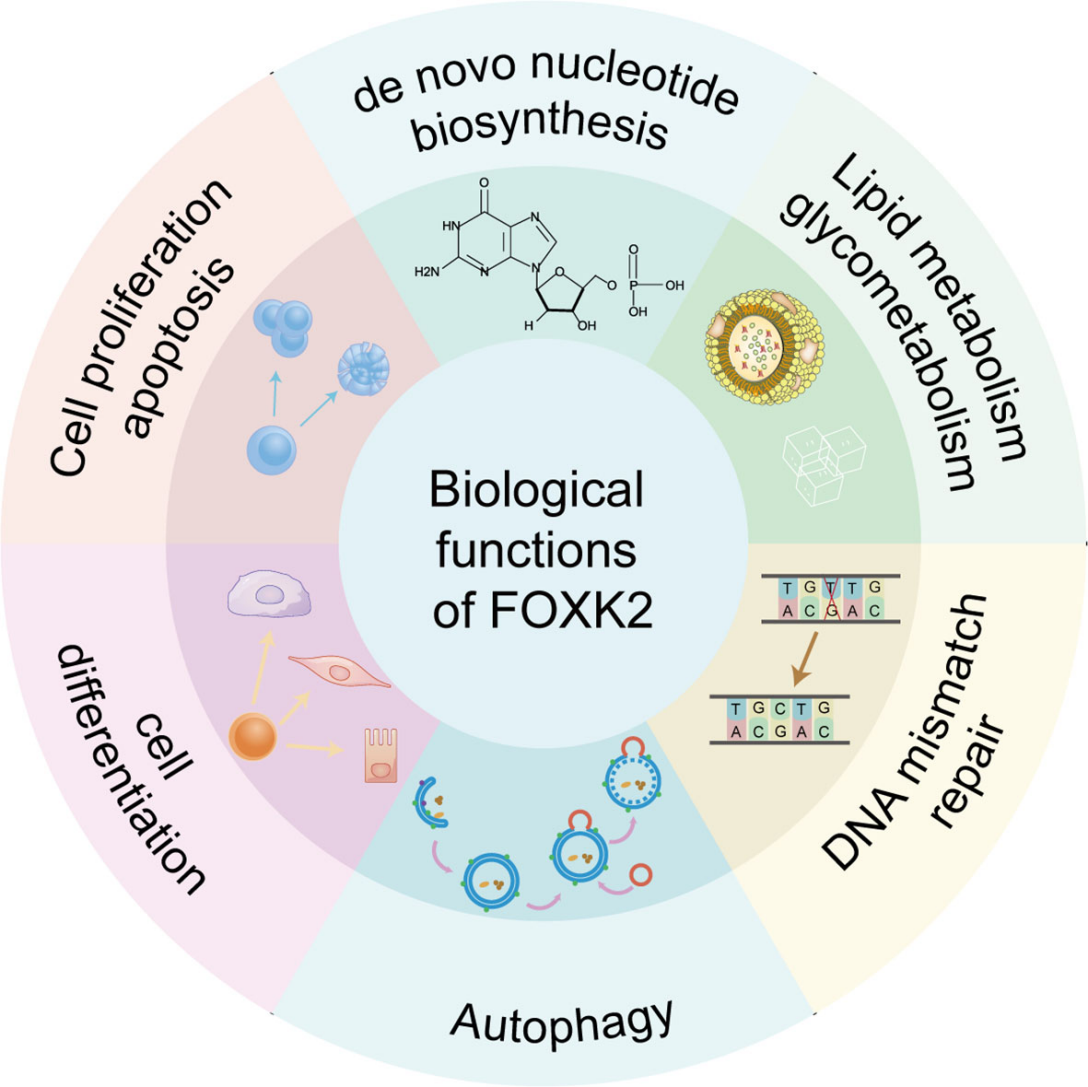
Biological functions of FOXK2. FOXK2 is involved in several important biological processes, including *de novo* nucleotide biosynthesis, lipid metabolism and glycometabolism, DNA mismatch repair, autophagy, cell differentiation, cell proliferation and apoptosis.

FOXK1 and FOXK2, the two members of the FOXK subfamily, have gained attention only in recent years, particularly in the context of tumors and metabolic diseases (2, 11). They are encoded by two distinct genes: FOXK1 (myonuclear factor, MNF) is localized on human chromosome 7p22.1 (12), encoding a 733 amino acid protein, while FOXK2 is located in 17q25.3, it was initially identified as an interleukin-enhancer binding factor (ILF) containing 660 amino acids (13, 14). They share an overall 56% identity (66% similarity), exhibit ubiquitous expression with highly conserved structure, and show obvious redundancy in function. FOXK proteins feature a forkhead-associated domain (FHA) in the N-terminal region in addition to the DBD domain that binds specifically to the evolutionarily conserved promoter region sequence GTAAACA (15) (**Figure 1B**). The FHA domain is essential for the interactions with other proteins (16). An approximately 30 amino acids insertion into FOXK2’s FHA determines the specificities of these two members. While both proteins interact with Dishevelled (DVL) proteins (17), SIN3A (18), Sds3 (17, 19) and BRCA1-associated protein 1 (BAP1) (20), the FHA domain in FOXK1 is known to interact with SRF (21), FOXO4 and MEF2 (22), whereas the FHA in FOXK2 interacts with TBL1, RbAp48, MTA3 and CoREST (23). Similarities between FOXK1 and FOXK2 suggest that they share functional redundancy as transcription factors involved in governing cell metabolism and proliferation. However, their precise regulation under distinct physiological and pathological conditions remains an intriguing area of investigation. Besides their classical molecular functions, such as *de novo* nucleotide synthesis (24), metabolic-related enzyme expression (25), and DNA mismatch repair (26), recent studies have revealed that FOXK2 plays a pivotal role in cell proliferation (27), differentiation (28), apoptosis (29) and autophagy (30) (**Figure 2**). Dysfunction of these FOXK2-involving processes is associated with the pathogenesis of various human diseases, including tumors and metabolic disorders. Therefore, this review summarizes the latest research on the structure, function, regulation, and impact of FOXK2 on human health, with potential implications for diagnosis and therapy.

## 2 Biological functions of FOXK2

### 2.1 *de novo* nucleotide biosynthesis

*De novo* nucleotides biosynthesis is crucial for tumor cell growth and metabolism, serving as a building block for DNA and RNA synthesis and as a source of ATP to fulfill the energy demands of rapidly proliferating tumor cells (31). The presence of FOXK2-binding motifs (GTAAACA) in the promoter region of various nucleotide biosynthesis genes suggests that FOXK2 plays a pivotal role in regulating nucleotide biosynthesis directly (24). Indeed, FOXK2 directly regulates the expression of nucleotide synthetic genes, promoting tumor growth and cancer cell resistance to chemotherapy. Notably, FOXK2 is SUMOylated by PIAS4, enabling its translocation into the nucleus, where it promotes the expression of nucleotide biosynthesis genes such as PRPS1, PPAT, CAD and PFAS. Conversely, when DNA damage is induced, as seen with agents like 5-fluorouracil (5-FU), SUMOylated FOXK2 levels significantly decrease, resulting in the loss of its capacity to promote nucleotide synthesis (24). Notably, since FOXK2 also regulates cellular metabolism (aerobic glycolysis) (32), it may also indirectly regulate *de novo* nucleotide synthesis, as cellular metabolism provides energy and materials for biosynthesis.

### 2.2 Lipid metabolism and glycometabolism

The metabolic regulation of FOXK2 has emerged as a novel and significant area of study in recent years. In the absence of insulin stimulation, FOXK2 is phosphorylated at S415/S419 by GSK3, resulting in its sequestration within the cytoplasm. Upon insulin stimulation, FOXK2 translocates from the cytoplasm to the nucleus via the AKT/mTOR pathway, where it participates in regulating insulin signaling and ultimately enhances the expression of genes associated with classical metabolic pathways, including glycolysis, glucose metabolism, fatty acid oxidation, and cholesterol biosynthesis. Both FOXK1 and FOXK2 work together synergistically to regulate mitochondrial β-oxidation by controlling the expression of key target genes, such as components of NADH dephosphorylase 1 subunit complex (Ndufs8, Ndufb3, Ndufa11, Ndufv1, Ndufv9, Ndufc2, and Ndufb7), ATPase H^+^ transforming subunits (Atp6v1g1, Atp6v1b2, Atp6v1f, Atp6v0b, Atp6v0a1, Atp6v0a2, and Atp6v0a4), cytochrome c oxidase subunits (Cox6b1, Cox7a1, Cox10, and Cox6b1) (25). Consistently, FOXK1 and FOXK2 upregulate the expression of phosphofructokinase, hexokinase-2, lactate dehydrogenase, and pyruvate kinase by directly binding to the promoter of these target genes, thereby inducing aerobic glycolysis (32). Furthermore, FOXK1 and FOXK2 enhance the activities of pyruvate dehydrogenase kinases 1 and 4 (PDK1 and PDK4), which facilitate the conversion of pyruvate to lactate, impeding further oxidation in mitochondria. Strikingly, FOXK1 and FOXK2 also modulate the expression of glutamate dehydrogenase 1 (GLUD1), indicating their involvement in the adaptive regulation of metabolic response to fasting or nutrient deprivation (32). Notably, FOXK1 has been demonstrated to regulate the glycolytic regulator HIF1α in a mTOR-dependent manner in fibroblasts *in vitro (* 33). However, administration of the mTOR inhibitor (rapamycin) has no effect on the regulation of FOXK2 on glycolysis in fully differentiated adipocytes, highlighting a distinction between FOXK1 and FOXK2. This finding supports the conclusion that FOXK2 regulates aerobic glycolysis by directly binding to the promoters of its target genes rather than via the mTOR pathway.

### 2.3 DNA mismatch repair

Although FOXK2 does not contain a catalytic domain for DNA repair, it has been found to have a higher affinity for G/T mismatched DNA compared to the consensus motif GTAAACA in the promoter region of target genes. This triggers a mechanism of DNA repair and prevents mutations (26). Some researchers propose that FOXK2 may recruit DNA repair proteins via its FHA domain to rectify the DNA mismatches and prevent the accumulation of additional mutations (26). This crucial role in DNA mismatch repair might contribute to the FOXK2 deficiency-induced cell death. However, further investigations are required to validate this hypothesis.

### 2.4 Autophagy

FOXK2 regulates autophagy through different mechanisms. As a transcriptional repressor of autophagy, FOXK2 enters the nucleus in response to adequate nutrition and recruits the Sin3A-HDAC co-repressor complex, reducing acetylated forms of histone H4 (H4ac), resulting in changes in nucleosome structure and restricting the transcription of key autophagy genes (18). Conversely, under starvation conditions, FOXK2 is transported from the nucleus to the cytoplasm by mTOR dependent phosphorylation, releasing its restriction on autophagy genes. Likewise, in response to DNA damage, FOXK2 can be phosphorylated through the ataxia-telangiectasia mutated (ATM)/checkpoint kinase 2 (CHK2) pathway, leading to the recruitment of 14-3-3 protein and sequestration in the cytoplasm, thereby inducing autophagy (30). Although both starvation and injury trigger autophagy by maintaining FOXK2 in the cytoplasm, starvation fails to activate the ATM/CHK2/FOXK2 signaling pathway (30), distinguishing two independent molecular mechanisms.

### 2.5 Cell differentiation

FOXK2 regulates cell differentiation by binding to numerous regulatory regions in human embryonic stem cells (HESCs). This binding serves as a pre-marking mechanism for regions that will be activated during cell differentiation (28). For instance, during the differentiation of ESCs into neural precursor cells (NPCs), the level of activated histone was significantly increased within FOXK2-prebound enhancer regions. Importantly, the binding of FOXK2 to regulatory regions is dynamic and reversable during cellular differentiation. For instance, while its binding to genes involved in neuronal development may diminish, it may rebind to regulatory regions of genes driving other developmental processes, such as mesoendoderm development (28).

### 2.6 Cell proliferation and apoptosis

FOXK2’s regulatory role in cell proliferation and apoptosis has become the subject of multiple studies in recent years. A FOXK1 deficient mice model demonstrated a severely runted phenotype and impaired skeleton muscle, linking FOXK1 to cell proliferation and differentiation (34). Subsequently, Anett Marais et al. demonstrated that FOXK2 is subject to control by the cell cycle-regulated protein kinases (CDK1-cyclinB as the major kinase complex), as its phosphorylation level exhibits a periodic rhythm during the cell cycle (35). Phosphorylated FOXK2 relieves the repression on the expression of p21, a key cell cycle regulator, thereby promoting cell cycle progression. Thus, FOXK2 behaves as a repressor in cell proliferation, and phosphorylation releases the suppression. Mutations in the two CDK phosphorylation sites of FOXK2, S368 and S423, have been found to induce cell apoptosis (35), yet the precise underlying mechanism remains elusive.

On the other hand, in 2015, another study proposed an activator role of FOXK2 in regulating cellular proliferation (27). This study demonstrated that knocking down FOXK2 reduces the expression of key markers for cell proliferation, such as BrdU incorporation and H3 phosphorylation, leading to decreased numbers of cells in the S and M phases. Additionally, in the absence of growth factors, knocking down FOXK2 induces caspase 3 cleavage and cell death through a Bcl-2-dependent pathway, implying a repressor role of FOXK2 in apoptosis. Notably, the downregulation of FOXK2 triggers compensatory activation of the mTOR signaling pathway, as evidenced by the phosphorylation of p70S6K and the upregulation of Growth Arrest and DNA-Damage 45s (Gadd45s). This compensatory mechanism helps mitigate the impact of imbalanced FOXK2 on cell apoptosis (27). Taken together, the role of FOXK2 in cell proliferation and apoptosis involves multiple pathways, and the microenvironment in different cell settings determines its actual function. Thus, further studies are required to elucidate its complexity.

## 3 The regulation to FOXK2

### 3.1 Transcriptional regulation of FOXK2

**Figure 3:**
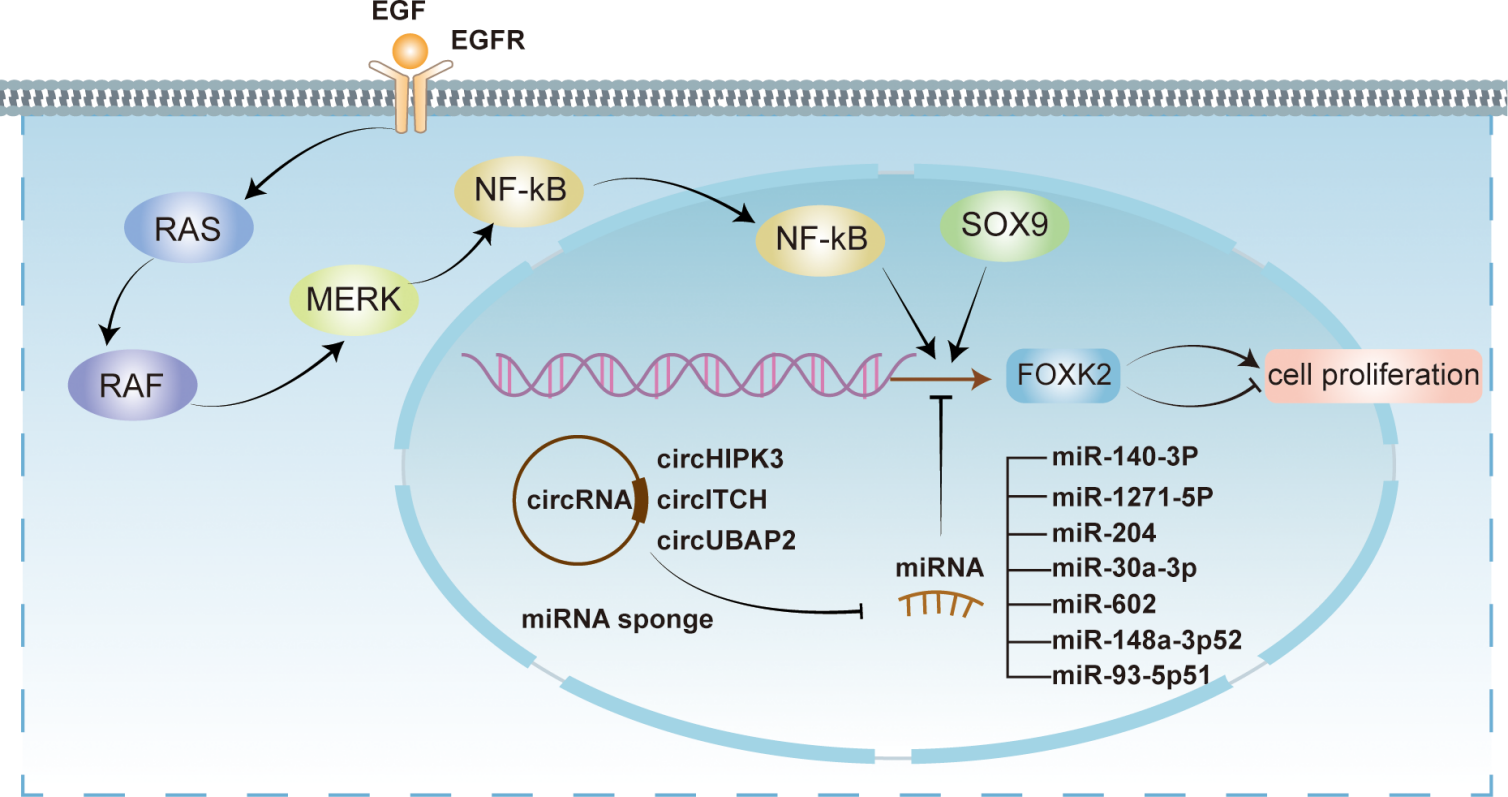
Transcriptional regulation of FOXK2. FOXK2 is regulated at transcriptional level by multiple mechanisms. Several miRNAs, including miR-140-3P, miR-1271-6P, miR-204, miR-30a-3p, miR-602, miR-148a-3p52, and miR-93-5p51 exert inhibitory effects on FOXK2 transcription. However, circRNAs such as circHIPK3, circITCH, and circUBAP2 act as miRNA sponges, relieving their inhibitory effects on FOXK2. Conversely, transcriptional activation of FOXK2 can be facilitated by SOX9 and EGF. EGF activates intracellular signaling pathways, including NF-kB pathway, ultimately leading to the activation of FOXK2 transcription through recognition of EGFR. Notably, the transcriptional regulation of FOXK2 can elicit both promotive and suppressive impacts on cellular proliferation.

FOXK2’s activity can be modulated at both transcriptional and post-translational levels. MicroRNAs (miRNAs), small non-coding RNAs, play a crucial role in regulating FOXK2 expression at the transcriptional level (**Figure 3**). As FOXK2 exhibits dual roles in different cell settings, miRNA-mediated regulation of FOXK2 also displays suppression or activation in cell proliferation in specific cell type. For instance, miR-140-3p has been shown to inhibit FOXK2 expression, promoting cell proliferation and angiogenesis in endothelial cells (36). Meanwhile, down-regulation of miR-1271-5p alleviates FOXK2 inhibition in hepatocellular carcinoma cells, facilitating tumor growth and metastasis through the PI3K/AKT signaling pathway (37). Similarly, miR-204 directly targets FOXK2 to suppress cell proliferation via the PI3K/AKT/mTOR pathway while promoting apoptosis in chicken atrophic ovarian cells (38). It is worth noting that circular RNAs (circRNAs) act as miRNA sponges to relieve FOXK2 inhibition. For instance, circHIPK3 sequesters miR-30a-3p to enhance FOXK2 expression, promoting fibroblast activation and glycolysis (39). Conversely, epigenetic mechanisms can activate miRNAs that indirectly hinder FOXK2 function. For example, hypomethylation near the promoter of miR-602 can trigger its expression, leading to the inhibition of FOXK2 and subsequently stimulating the proliferation and metastasis in esophageal squamous cell carcinoma (ESCC) (40).

In addition to miRNA, other transcriptional activation regulators (**Figure 3**), such as epidermal growth factors (EGFs), can activate FOXK2 expression through the extracellular signal-regulated kinase (ERK)/nuclear factor κB (NF-κB) pathway (41). Furthermore, SOX9, another regulatory factor, can bind to the promoter sequence of FOXK2 and activate its transcriptional expression, directly participating in the initiation and progression of colorectal cancer (42).

### 3.2 Post-translational regulation of FOXK2

**Figure 4:**
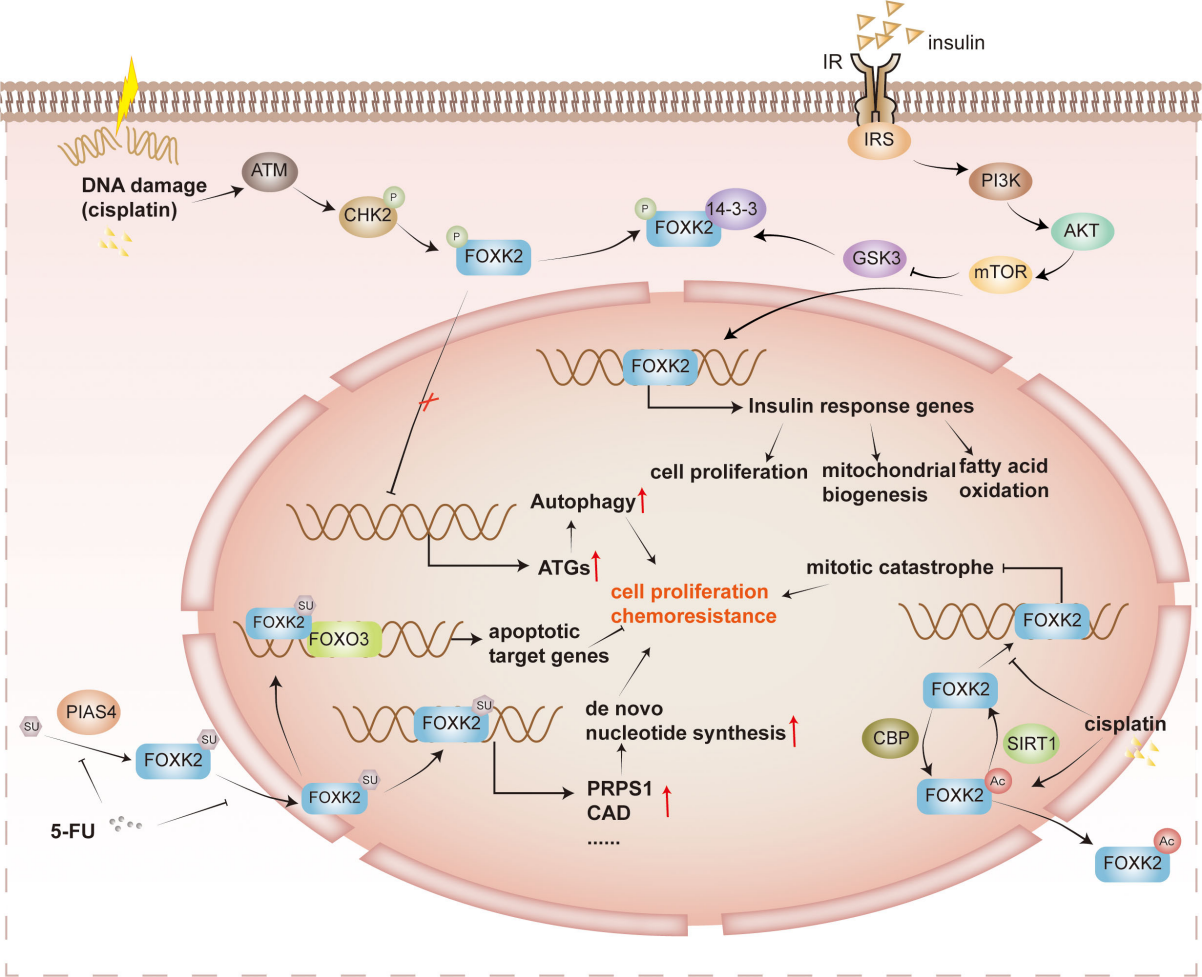
Post-translational regulation of FOXK2. Cisplatin-induced DNA damage and various other factors activate the protein ATM, triggering the phosphorylation of CHK2. Phosphorylated CHK2 further enhances the phosphorylation of FOXK2. However, phosphorylated FOXK2 binds to 14-3-3 proteins in the cytoplasm, preventing its translocation into the nucleus. As a result, the inhibition on ATGs is lifted, leading to tumor cell survival and drug resistance. In addition, insulin signaling, specifically via the PI3K/AKT/mTOR pathway, plays a role in inhibiting the phosphorylation of GSK3 on FOXK2. This inhibition facilitates the nuclear translocation of FOXK2, promoting the expression of insulin response genes involved in cell proliferation, mitochondrial biosynthesis, and fatty acid oxidation. FOXK2 is also regulated by SUMOylation. PIAS4 catalyzes the SUMOylation of FOXK2, which facilitates its entry into the nucleus, and promotes *de novo* nucleotide synthetic gene expression, such as PRPS1 and CAD. In the context of tumor proliferation and drug resistance, 5-FU inhibits the SUMOylation of FOXK2, reducing tumor cell proliferation and drug resistance. FOXK2 undergoes acetylation catalyzed by CBP, leading to its translocation to the cytoplasm. However, SIRT1 catalyzes the deacetylation reaction, enabling FOXK2 to bind more effectively to the target promoter region. This regulation of gene expression and modulation of mitotic catastrophe ultimately contribute to tumor proliferation and drug resistance. It’s worth noting that cisplatin can also promote the acetylation and translocation of FOXK2 into the cytoplasm, affecting its function.

Post-translational modifications are ubiquitous and crucial for regulating protein localization or activity. FOXK2 is also subject to post-translational modifications, including phosphorylation, SUMOylation, and acetylation (**Figure 4**).

#### 3.2.1 Phosphorylation

Several kinase pathways are involved in phosphorylating FOXK2 and lead to distinct outcomes. Previous studies have shown that FOXK2 phosphorylation fluctuates during the cell cycle, peaking in the M phase. The CDK/cyclin complex catalyzes FOXK2 phosphorylation at S368 and S423, affecting its protein stability and transcriptional activity (35). In response to DNA damage, ATM promotes the phosphorylation of CHK2 at Thr68, enhancing its interaction with FOXKs. CHK2 then phosphorylates FOXK1 at S130 and FOXK2 at S61, facilitating their binding to 14-3-3 proteins. The binding of 14-3-3 sequesters FOXKs in the cytoplasm, releasing their inhibition on autophagy-related gene (ATG) expression and consequently enhancing autophagy (30). Furthermore, GSK3 phosphorylates FOXK2 at S415/S419, resulting in its sequestration within the cytoplasm without insulin stimulation (25). In summary, FOXK2 phosphorylation is tightly regulated by various kinase pathways throughout the cell cycle and in response to DNA damage, leading to distinct cellular outcomes such as transcriptional regulation and modulation of autophagy.

#### 3.2.2 SUMOylation

FOXK2 is SUMOylated at K527 and K633 by PIAS4 (24, 43). This modification triggers the translocation of FOXK2 into the nucleus, where it can be recruited to target gene promoters and activate transcription. For instance, binding to the promoter of the tumor suppressor FOXO3 mediates paclitaxel-induced cytotoxicity in breast cancer cells (44), while binding to the promoter of *de novo* nucleotide biosynthesis genes facilitates hepatocellular carcinoma growth and chemotherapy resistance (24).

#### 3.2.3 Acetylation

FOXK2 undergoes acetylation by cAMP response element binding protein (CBP) and deacetylation by sirtuin 1 (SIRT1) at K223 (45). In the absence of cisplatin stimulation, FOXK2 remains hypoacetylated in the cell nucleus, impeding mitotic catastrophe and diminishing tumor cell apoptosis. However, cisplatin can disrupt the interaction between SIRT1 and FOXK2, leading to an increase in FOXK2 acetylation. Hyperacetylated FOXK2 exhibits reduced nuclear distribution, significantly affecting cell cycle-related genes, leading to cell cycle arrest and promoting apoptosis of cancer cells. Ultimately, this enhances tumor cell sensitivity to cisplatin (45). Acetylation may serve as a general mechanism to regulate FOX protein’s subcellular localization. For instance, FOXO3 can also be deacetylated by SIRT1, and it collaborates with FOXK2 to regulate the apoptosis of cancer cells (46). In contrast to FOXK2, SIRT1 deacetylates FOXO3 and mediates the nuclear export of FOXO3, which relieves the induction of apoptosis by FOXO3.

### 3.3 Regulation by protein-protein interaction

#### 3.3.1 Synergistic inhibition

**Figure 5:**
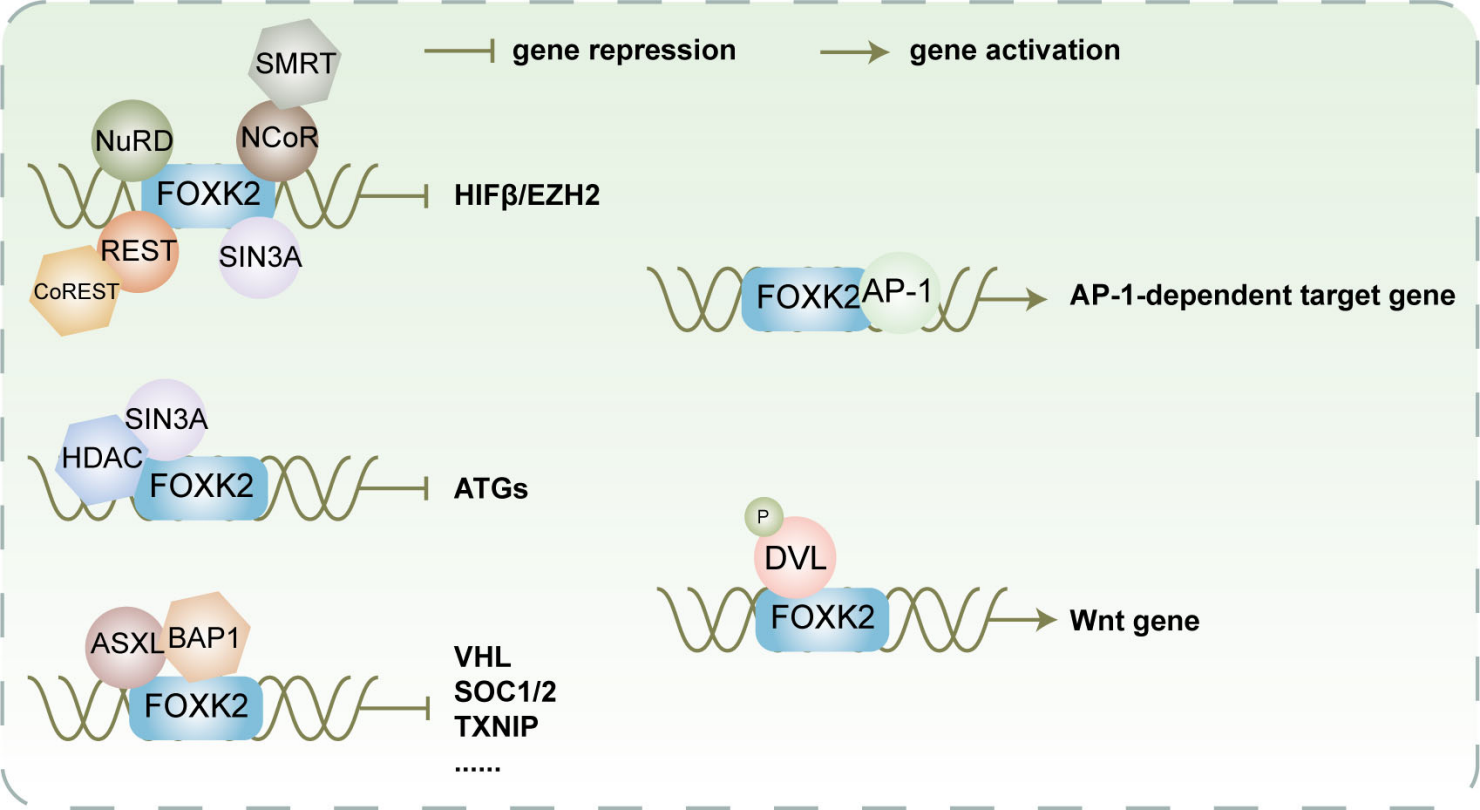
FOXK2 facilitates recruitment of various co-inhibitory or co-activating complexes for transcriptional regulation of its downstream target gene FOXK2 recruits NuRD, SIN3A, NCoR/SMRT, and REST/CoREST to cooperatively impede the expression of HIFβ and EZH2. Additionally, it collaborates with SIN3A/HDAC to synergistically inhibit the expression of ATGs. Furthermore, FOXK2 recruits ASXL/BAP1 to collaboratively suppress the expression of VHL, SOC1/2, TXINP, and other genes participating in cellular metabolism and proliferation. In contrast, FOXK2 recruits AP-1 to activate the expression of AP-1-dependent target genes. Moreover, it recruits phosphorylated DVL to induce the expression of Wnt-related genes, ultimately activating the Wnt signaling pathway.

FOXK2 interacts with multiple co-inhibitory complexes (**Figure 5**), including NCoR/SMRT, SIN3A, NuRD, and REST/CoREST, to function as a transcriptional repressor. These complexes possess HDAC activity and modulate chromatin status through post-translational modifications, resulting in synergistic inhibition of a series of FOXK2-targeted hypoxia-related genes, including HIF1β and EZH2, effectively suppressing the hypoxic response (23). The recruitment of the Sin3A-HDAC co-inhibitory complexes to FOXK2 occurs under adequate nutrition conditions, resulting in alterations in nucleosome structure through inhibiting histone H4 acetylation and subsequently suppressing autophagy and atrophy-related genes. However, during starvation, dissociation of FOXK2 from chromatin leads to the loss of synergistic activity with Sin3A-HDAC, inducing autophagy (18).

In addition to binding co-inhibitory complexes with acetylation activity, FOXK2 also interacts with Polycomb repressive deubiquitinase (PR-DUB) complexes, which have BRCA1-associated protein 1 (BAP1) as the core component (47). Under physiological conditions, additional sex combs like 1 (ASXL1) can bind to BAP1 which subsequently binds to chromatin by interacting with FOXK2 (20, 47). This interaction modulates the chromatin microenvironment by regulating H2AK119 ubiquitination and controlling the expression of FOXK2 target genes, including Von Hippel-Lindau syndrome (VHL), suppressor of cytokine signaling 1 and 2 (SOCS1/2), thioredoxin interacting protein (TXNIP), and others (20). However, C-terminal truncated ASXL mutants are frequently observed in myeloid neoplasms. Although they can still bind BAP1 normally, they cannot bind FOXK2. Consequently, the PR-DUB complex cannot be recruited onto chromatin (20), leading to improper expression of downstream tumor suppressor genes of FOXK2, thereby promoting tumorigenesis.

#### 3.3.2 Synergistic activation

FOXK2 not only synergizes with co-repressor proteins, but also collaborates with co-activator proteins to activate downstream target genes (**Figure 5**). As discovered by Ji et al., the binding regions of FOXK2 commonly include AP-1 binding motifs TGA(G/C)TCA, suggesting that FOXK2 and AP-1 exhibit a synergistic effect on transcription (48). Mechanistically, FOXK2 may facilitate the recruitment of AP-1 major components FOS and JUN to chromatin by opening up the chromatin structure, thereby promoting AP-1-dependent target gene expression. Additionally, FOXK1 and FOXK2 are capable of binding to the PDZ domain-localized region (residues 250 to 355) of phosphorylated DVL through a hydrophobic motif in the FHA domain (L137-F145-F154), facilitating their interaction (17). Upon induction of Wnt signaling, CK1 and MARK kinases catalyze DVL phosphorylation, leading to its translocation into the nucleus where it interacts with FOXKs to promote transcriptional activation of downstream genes. Moreover, DVL can transduce Wnt signaling to the GSK3/β-destruction complex and inhibit its activity, promoting β-catenin stabilization (17). The formation of the β-catenin/TCF/cJun transcription complex is facilitated by FOXK2 and DVL under transcriptional regulation, enhancing Wnt/β-catenin signaling (49).

## 4 FOXK2 and diseases

### 4.1 FOXK2 and cancers

An increasing number of studies have revealed the intricate role of FOXK2 in tumors, as it can have either inhibitory or promoting effects. For instance, reduced expression of FOXK2 has been linked to tumor suppression in breast cancer (23, 50), cervical cancer (51), glioma (52), gastric cancer (53), ESCC (40), non-small cell lung cancer (NSCLC) (54), and clear-cell renal cell carcinoma (ccRCC) (29), while FOXK2 expression was significantly upregulated in colorectal cancer (17, 41, 42), ovarian cancer (55) and hepatocellular carcinoma (37, 56) (summarized in **Table 1**). The following sections will elaborate on the roles of FOXK2 in promoting or inhibiting different types of tumors.

**Table 1:** The expression, function, and involved pathways of FOXK2 in different types of cancer.

Type of cancer	FOXK2 expression	Function	Pathway
Hepatocellular carcinoma (37)	High	Promote tumor proliferation/metastasis	MiR-1271-5p/FOXK2/PI3K/AKT
Colorectal cancer (42)	High	Promote tumor development	SOX9/FOXK2
Colorectal cancer (17)	High	Promote tumor proliferation/metastasis	DVL/FOXK2/Wnt/β-catenin
Colorectal cancer (41)	High	Promote invasion and metastasis	EGFR/ERK/NF-κB/FOXK2/EGFR and ZEB1
Breast cancer (23)	Low	Inhibit tumorigenesis and invasion	FOXK2/Co-inhibitory complex (NCoR/SMRT, SIN3A, NuRD, and REST/CoREST)/HIF1β and EZH2
Ovarian cancer stem cells (55)	Low	Inhibit endoplasmic reticulum (ER) stress	FOXK2/ERN1/IRE1α/UPR
Myeloid tumors (47)	Low	Maintain cellular metabolism and homeostasis	ASXL1/BAP1/FOXK2/tumor suppressor genes (ZNF516, MAGI1, SOCS1/2, TXNIP and VHL)
Non-small cell lung cancer (54)	Low	Inhibit metastasis	FOXK2-PI3K/AKT
Non-small cell lung cancer (54)	Low	Inhibit proliferation	FOXK2/CDK4 and cyclin D1
Cervical cancer (51)	Low	Tumor suppression	Circ-ITCH/miR-93-5p51/FOXK2
Clear-cell renal cell carcinoma (57)	Low	Tumor suppression	Circ-UBAP2/miR-148a-3p52/FOXK2
Esophageal squamous cancer (40)	Low	Inhibit proliferation and metastasis	Methylation/miR-602/FOXK2
Gastric cancer (53)	Low	Tumor suppression	Unknown
Glioma (52)	Low	Tumor suppression	Unknown

#### 4.1.1 Tumor proliferation, invasion and metastasis

FOXK1 and FOXK2 have been linked to proliferation and metastasis in human colorectal cancer, which is associated with DVL nuclear localization and activation of the Wnt/β-catenin signaling pathway, the latter plays a pivotal role in regulating cell proliferation and differentiation, self-renewal, tissue homeostasis as well as embryonic development (58, 59). The Wnt signal triggers DVL phosphorylation, leading to its translocation into the nucleus and interaction with FOXK2 to facilitate transcriptional activation of the β-catenin/TCF/cJun complex, thus reactivating Wnt/β-catenin signaling (17).

Protein quality and cellular homeostasis are critical for cancer survival and progression. FOXK2 participates in endoplasmic reticulum (ER) stress control to promote the maintenance of stemness and tumor progression in ovarian cancer stem cells (CSCs) (55). It binds to the distal region of the second intron of the unfolded protein response (UPR) sensor protein IRE1α and directly regulates its expression. IRE1α possesses both endonuclease and kinase activities; it splices the mRNA encoding the transcription factor X-box binding protein 1 (XBP1). The production of an XBP1 splice variant exhibits potent transcriptional activity and confers protection against apoptosis induced by ER stress (60). In cancer cells, the UPR pathway is usually activated, probably related to metabolic stress caused by accelerated nucleotide synthesis and cell proliferation, highlighting the role of FOXK2 metabolic regulation and protein homeostasis for cancer development.

In human colorectal cancer, FOXK2 also exhibits a positive feedback loop as a regulatory factor in the cell cycle and tumor progression. EGF binds to EGFR on the cell surface and activates FOXK2 expression via the extracellular signal-regulated kinase (ERK)/NF-κB pathway. Subsequently, FOXK2 activates the transcription of EGFR and ZEB1, crucial regulators of the epithelial-mesenchymal transition (EMT), invasion, and metastasis in colorectal cancer (41). Notably, FOXK2’s regulation on EGFR can be context-dependent and cell-specific; it functions as a tumor suppressor gene in ccRCC by inhibiting EGFR and inducing apoptosis (29), contrasting its role in colorectal cancer (41).

Furthermore, FOXK2 is significantly upregulated in hepatocellular carcinoma (HCC) cells. Studies have demonstrated that elevated FOXK2 can enhance Snail expression and reduce E-cadherin levels, promoting EMT in HCC cells (56). This process is closely associated with PI3K/AKT signaling pathway activation.

In summary, FOXK2 emerges as a potential biomarker for certain tumors in which it plays a promoting role, indicating its utility in guiding tailored tumor treatment strategies. By identifying FOXK2 expression patterns, clinicians can better understand the underlying mechanisms driving tumor progression and select more personalized therapeutic approaches for patients. This highlights the importance of FOXK2 as not only a diagnostic marker but also a potential therapeutic target in cancer management.

#### 4.1.2 Cancer suppression

FOXK2 plays a regulatory role in hypoxia pathway, prevalently in locally advanced solid tumors, and holds significant pathological and physiological effects in tumor progression and invasion (61). FOXK2 interacts with various co-suppressor transcriptional complexes including NCoR/SMRT, SIN3A, NuRD, and REST/CoREST, to inhibit multiple hypoxia response genes such as HIF1β and zeste homolog 2 (EZH2) (23). In breast cancer cells, FOXK2 is positively regulated by estrogen receptor alpha (ERα). However, FOXK2 also plays a role in the degradation of ERα through the ubiquitin E3 ligase BRCA1/BARD1 complex (50). As breast cancer progresses, there is a gradual loss of FOXK2 expression, leading to the activation of the hypoxia pathway and an increase in the expression of enhancer of EZH2. Notably, the hypoxia signal further increases the level of EZH2, down-regulating FOXK2. This intensifies the activation of the hypoxia pathway, promoting EMT and metastasis in breast cancer (23). Elevated expression of EZH2 serves as a significant hallmark of invasive breast cancer (62). In general, low FOXK2 expression correlates with higher histological grade, positive lymph nodes, and ERα^-^/PR^-^/HER2^-^ negativity.

In myeloid tumors, ASXL1 C-terminal truncated mutants are common (63). The C-terminal truncated ASXL1 mutant protein retains its binding ability to BAP1, but loses its interaction with multiple DNA-binding transcriptional regulators, including FOXK1 and FOXK2 (20). As a result, multiple tumor suppressor genes, such as zinc finger protein 516 (ZNF516), membrane-associated guanylate kinase 1(MAGI1), SOCS1/2, TXNIP and VHL, are down-regulated, ultimately promoting cancer development and disrupting cellular metabolism and homeostasis (20).

In NSCLC cells, FOXK2 inhibits EMT through the PI3K-Akt pathway, contrasting with its promoting role in hepatocellular carcinoma cells (54). It suppresses CDK4 and cyclin D1 expression, arresting cells in the S phase, thus impeding NSCLC proliferation (54). Furthermore, FOXK2 expression is inversely correlated with gastric cancer (53) and glioma (52) grade as well as prognosis. However, further exploration is required to understand the regulatory mechanisms of FOXK2 in gastric cancer and glioma. Additionally, FOXK2 exerts its tumor suppressive effects through various circRNAs that sponge miRNAs and upregulate FOXK2 expression. For instance, Circ-ITCH (51) and circ-UBAP2 (57) function as tumor suppressors in cervical cancer and ccRCC, respectively, by sequestering miR-93-5p51 and miR-148a-3p52, thereby enhancing the expression of FOXK2. Remarkably, hypermethylation near the upstream promoter of miR-602 (40) negatively regulates its expression, upregulating FOXK2 to inhibit the invasion and metastasis of ESCC.

The dual role of FOXK2 reveals the intricate complexity and specificity of its involvement in tumor development and progression. Understanding these nuances is crucial for elucidating the precise role of FOXK2 in different cancer types and for developing targeted therapeutic strategies. Moreover, it underscores the need for further research to unravel the intricate molecular mechanisms underlying FOXK2’s function in cancer, ultimately leading to more effective treatments and improved patient outcomes.

#### 4.1.3 FOXK2 and chemoresistance

Cancer cells often develop drug resistance through protective mechanisms, such as damage-induced autophagy (30). Autophagy is a cellular process that maintains homeostasis by removing misfolded proteins and damaged organelles in response to various stressors (64). FOXKs act as transcriptional repressors in autophagy (18), contributing to chemotherapy resistance in cancer cells. Specifically, the ATM enzyme promotes phosphorylation of CHK2 at T68, enhancing interactions between CHK2 and FOXKs. Subsequently, CHK2 phosphorylates FOXK1 and FOXK2 at S130 and S61, respectively, sequestering them in the cytoplasm through binding with 14-3-3 proteins (**Figure 4**). This cytoplasmic localization alleviates ATGs inhibition, promoting autophagy (30). Hyperphosphorylation of FOXKs, caused by cancer-derived mutations, promotes autophagy and chemoresistance. However, combining the autophagy inhibitor chloroquine (CQ) with chemotherapy drugs, such as cisplatin (30), effectively overcomes chemoresistance induced by FOXKs mutants.

Another factor contributing to chemotherapy resistance is the abnormal activation of the *de novo* nucleotide synthesis pathway, crucial for rapid tumor proliferation (**Figure 4**) (24). A FOXK2-specific binding motif and a SUMO-related signal exist within the promoter region of genes governing nucleotide biosynthesis. PIAS4 catalyzes FOXK2 SUMOylation at K527 and K633 (24, 43), facilitating nuclear translocation of FOXK2 and promoting *de novo* nucleotide synthesis, leading to hepatocellular carcinoma resistance against the chemotherapeutic drug 5-FU (24). Notably, FOXK2 SUMOylation can be inhibited by DNA damage induced by chemotherapy and radiation, presenting an opportunity to improve treatment efficacy by combing chemotherapy drugs with *de novo* nucleotide synthesis inhibitors, such as mycophenolate mofetil, MMF (24).

FOXK2 SUMOylation also plays a role in breast cancer treatment. SUMOylation facilitates its recruitment to the forkhead response elements (FHRE) region the FOXO3 promoter, activating downstream apoptotic target genes and enhancing paclitaxel cytotoxicity (44). However, in paclitaxel-resistant cells, FOXK2 accumulates in the nucleus but lacks SUMOylation, hindering recruitment FOXO3 for downstream gene activation, contributing to cellular resistance. Both PIAS4-mediated SUMOylation and CHK2-mediated phosphorylation of FOXK2 can modulate its nuclear localization and function. The two modification sites are spatially distant and do not interfere with each other during the regulatory process, thus they synergistically control the perception of DNA damage signals.

Additionally, SIRT1 catalyzes FOXK2 deacetylation, reducing tumor cell apoptosis and decreasing sensitivity to the chemotherapy drug cisplatin (**Figure 4**) (45). However, cisplatin stimulation inhibits SIRT1 and FOXK2 interaction, leading to increased acetylation levels at K223 of FOXK2, enhancing sensitivity to chemotherapy drugs. Therefore, the combined application of cisplatin and SIRT1 inhibitors offers promising prospects for developing novel cancer chemotherapy strategies (45).

#### 4.1.4 FOXK2 and tumor metabolism, tumor immune and tumor microenvironment

FOXK2 emerges as a potent regulator of lipid oxidation and aerobic glycolysis (25, 32). It undergoes translocation from the cytoplasm to the nucleus via the AKT/mTOR pathway, where it participates in the regulation of lipid oxidation related gene expression (25). Moreover, FOXK2 regulates the expression of genes involved in aerobic glycolysis by binding to their promoters, including phosphofructokinase, hexokinase-2, lactate dehydrogenase, and pyruvate kinase (32). Through regulation of glucose and lipid metabolism, FOXK2 is closely linked to tumor initiation and progression. It is worth noting that tumor cells significantly contribute to remodeling the tumor microenvironment (TME) by producing large amounts of lactate through aerobic glycolysis (65) and enhancing lipid uptake and oxidation (66). This TME remodeling, in turn, influences the metabolic profile and immunophenotype of immune cells, leading to their immunosuppression (67). For instance, regulatory T (Treg) cells metabolize lactate to support their proliferative and suppressive functions, and tumor-infiltrating Treg cells require lactate uptake to maintain their heightened suppressive function (68). Additionally, up-regulation of lipid uptake and FAO elevates lipid metabolism in tumor-associated macrophages (TAMs), Tregs and myeloid-derived suppressor cells (MDSCs), thereby promoting their immunosuppressive function (66). However, no direct association between FOXK2 and the tumor microenvironment or immune response has been reported thus far; further investigation is warranted.

### 4.2 FOXK2 and metabolic disorders

Recent studies have highlighted FOXK2’s role in metabolic disorders, particularly in promoting glycolysis and contributing to pulmonary fibrosis through the circHIPK3/miR-30a-3p/FOXK2 pathway (39). Mechanistically, transforming growth factor-β1 (TGF-β1), a key growth factor for fibroblast activation (69), triggers glycolytic reprogramming (70) by inducing the expression of circHIPK3 in fibroblasts. The sponge effect of circHIPK3 on miR-30a-3p restricts its expression and ultimately activates FOXK2. FOXK2 can promote fibroblast glycolysis and activation, which is also the main driver of pulmonary fibrosis. Targeting circHIPK3/FOXK2 pathway can effectively alleviate TGF-β1 or silica-induced pulmonary fibrosis (39).

Furthermore, SOX9 acts as a master regulator of cardiac fibrosis and inflammation in fibroblasts. Considering that SOX9 upregulates FOXK2 expression by directly binding to the FOXK2 promoter (42), it is speculated that FOXK2 may play a role in SOX9-mediated cardiac fibrosis or other cardiomyopathies. The targeted deletion of SOX9 in fibroblasts significantly reduces the proliferation and contraction ability of fibroblasts, leading to improved left ventricular dysfunction and myocardial scarring induced by myocardial infarction (71). The precise involvement of FOXK2 in these disorders warrants further investigation.

Additionally, FOXK2’s involvement in intracellular insulin signaling via the AKT/mTOR pathway (25) suggests its potential role in regulating various metabolic pathways such as glycolysis and cholesterol biosynthesis, which are crucial in diabetes and other metabolic disorders (25). Aberrant FOXK2 function may contribute to the dysfunction of these pathways and impair sensitivity to insulin stimulation in diabetic patients. Further exploration is required to uncover FOXK2’s impact on these pathways and its potential implications for insulin sensitivity in diabetic patients.

## 5 Conclusion and prospect

FOXK2 has emerged as a significant player in various physiological and pathological processes, including *de novo* nucleotide biosynthesis, glycolysis, fatty acid β-oxidation, DNA damage repair, autophagy, cell proliferation and differentiation, as well as apoptosis. It has been demonstrated to behave as a transcriptional repressor or activator in distinct cell settings. Dysregulation of FOXK2 has been linked to the development of cancers and other metabolic disorders. Consequently, further investigations into the underlying mechanism in FOXK2 dysregulation and potential therapeutic strategies are imperative.

The pivotal role of FOXK2 in the development of various tumors, along with its distinct expression patterns in normal and tumor tissues, suggests the potential clinical application of FOXK2 as a biomarker for the diagnosis and prognosis of different malignant tumors. For instance, during breast cancer progression, there is a progressive loss of FOXK2 expression, which correlates closely with poor prognostic indicators such as higher histological grade, lymph node positivity, and ERα^−^/PR^−^/HER2^−^ status, highlighting its potential as a prognostic biomarker (23). As FOXK2 regulates DVL and Wnt/β-catenin signaling pathway, makes it a potential target for cancer progression and metastasis. While no studies on inhibitors targeting FOXK2 have been reported so far, inhibitors targeting other members of the FOX family have shown effectiveness in treating tumors. For example, a cell-penetrating ARF peptide was identified as an inhibitor of FOXM1, it can be utilized as a practical treatment method to reduce proliferation and induce apoptosis in liver cancer cells within mouse tumor models (72). Sorafenib and paclitaxel, common anticancer drugs used for liver cancer treatment, have also been proven to inhibit liver cancer cell proliferation by targeting FOXM1 (73, 74). Additionally, a 15-mer synthetic peptide, P60, acts as an inhibitor of FOXP3, impairing Treg cell activity while improving vaccine efficacy in mice (75). Given FOXK2’s high expression in a variety of tumors and its close association with tumor proliferation and metastasis, developing FOXK2-specific inhibitors may provide new valuable therapeutic strategies for tumor treatment.

Notably, considerable progress has been achieved in understanding the mechanisms of chemotherapeutic resistance involving FOXK2. For instance, the PIAS4-catalyzed SUMOylation promotes *de novo* nucleotide biosynthesis in the nucleus, a process crucial for HCC cells’ resistance to 5-FU. Therefore, co-administration of 5-FU and MMF may potentiate the cytotoxicity of 5-FU against HCC cells. However, scientific challenges persist, such as further investigating the potential correlation between nucleotide synthesis and glucose metabolism (aerobic glycolysis) regulated by FOXK2. Additionally, a more comprehensive understanding of how TGF-β1 enhances circHIPK3 expression is needed, along with a thorough exploration of FOXK2’s translocation between the nucleus and cytoplasm following phosphorylation and SUMOylation. Moreover, a comprehensive investigation of the intricate connections between FOXK2, Gadd45s induced by compensatory mTOR activation, and DNA mismatch repair is warranted. Finally, the *in vivo* functions of FOXK2 in tumor initiation and development still needs further in-depth studies using tissue-specific mouse models. Addressing these challenges will advance our understanding of FOXK2’s role in tumor formation, proliferation, invasion and metastasis, as well as in cancer therapy or drug resistance, eventually paving the way for targeting FOXK2 in the treatment of cancer and metabolic diseases.

## Author contributions

XX: Writing – original draft, Writing – review & editing. XQ: Writing – original draft. SZ: Writing – original draft. SW: Writing – original draft. QS: Writing – review & editing. YY: Writing – review & editing, Writing – original draft. PZ: Writing – original draft, Writing – review & editing.

## Glossary

**Table:**

FOXK	the Forkhead box
DBD	DNA binding domain
MNF	myonuclear factor
ILF	interleukin-enhancer binding factor
FHA	forkhead-associated domain
DVL	Dishevelled
BAP1	BRCA1-associated protein 1
PDK1 and PDK4	pyruvate dehydrogenase kinases 1 and 4
GLUD1	glutamate dehydrogenase 1
ATM	ataxia-telangiectasia mutated
HESCs	human embryonic stem cells
CHK2	checkpoint kinase 2
NPCs	neural precursor cells
Gadd45s	Growth Arrest and DNA-Damage 45s
circRNAs	circular RNAs
miRNAs	microRNAs
ESCC	esophageal squamous cell carcinoma
EGFs	epidermal growth factors
ERK	extracellular signal-regulated kinas
NF-κB	nuclear factor κB
ATG	autophagy-related gene
CBP	cAMP response element binding protein
SIRT1	sirtuin 1
PR-DUB	Polycomb repressive deubiquitinase
BAP1	BRCA1-associated protein 1
ASXL1	additional sex combs like 1
VHL	Von Hippel-Lindau syndrome
SOCS1/2	suppressor of cytokine signaling 1 and 2
TXNIP	thioredoxin interacting protein
NSCLC	non-small cell lung cancer
ccRCC	clear-cell renal cell carcinoma
ERK	extracellular signal-regulated kinase
EMT	epithelial-mesenchymal transition
CSCs	cancer stem cells
ER	endoplasmic reticulum
UPR	unfolded protein response
XBP1	X-box binding protein 1
HCC	hepatocellular carcinoma
Erα	estrogen receptor alpha
EZH2	enhancer of zeste homolog 2
ZNF516	zinc finger protein 516
MAGI1	membrane-associated guanylate kinase 1
CQ	chloroquine
FHRE	forkhead response elements
TGF-β1	transforming growth factor-β1
